# Multiple-Jet Needleless Electrospinning Approach via a Linear Flume Spinneret

**DOI:** 10.3390/polym11122052

**Published:** 2019-12-11

**Authors:** Liang Wei, Chengkun Liu, Xue Mao, Jie Dong, Wei Fan, Chao Zhi, Xiaohong Qin, Runjun Sun

**Affiliations:** 1 School of Textile Science and Engineering, Xi’an Polytechnic University, Xi’an 710048, China; liangwei@xpu.edu.cn (L.W.); liuchengkun@xpu.edu.cn (C.L.); maoxue@xpu.edu.cn (X.M.); dongjie619@126.com (J.D.); fanwei@xpu.edu.cn (W.F.); zhichao@xpu.edu.cn (C.Z.); 2 Key Laboratory of Textile Science & Technology, Ministry of Education, College of Textiles, Donghua University, Shanghai 201620, China; xhqin@xpu.edu.cn

**Keywords:** multiple-jet, spinneret, electric field, morphology, nanofiber, diameter

## Abstract

There is a great limitation to improving the quality and productivity of nanofibers through the conventional single-needle method. Using needleless electrospinning technology to generate multiple jets and enhance the productivity of nanofibers has attracted lots of interest for many years. This study develops a novel linear flume spinneret to fabricate nanofibers. Multiple jets with two rows can be formed simultaneously on the surface of the spinneret. The solution concentration has a significant impact on the average nanofiber diameter compared with applied voltage and collection distance. The effects of different spinning process parameters on the productivity of nanofibers are investigated. High-quality nanofibers with small nanofiber diameter and error can be fabricated successfully. The average nanofiber diameter is 108 ± 26 nm. The average error is 24%. The productivity of nanofibers can reach 4.85 ± 0.36 g/h, which is about 24 times more than that of the single-needle method. This novel linear flume spinneret needleless electrospinning technology exhibits huge potential for mass production of nanofibers in the field of industrialization.

## 1. Introduction

Nanofibers have attracted much attention for many years because of their outstanding properties. Compared with traditional fibers, nanofibers exhibit many advantages, such as high specific surface area, high porosity and controllable nanofiber diameter [1]. Nanofibers have been widely applied in many fields, such as filtration materials [2,3], sensor [4], tissue engineering scaffolds [5,6], sensors [7] and energy storage materials [8]. It is common knowledge that electrospinning is a simple and convenient method to prepare nanofibers. Nevertheless, some problems need to be addressed for further practical applications, such as improving the productivity of nanofibers and enhancing the quality of nanofibers [9]. The productivity of nanofibers with the conventional single-needle method is very low. How to improve the productivity of nanofibers is a significant research topic.

Many methods have been attempted to improve the productivity of nanofibers. A simple approach was to increase the number of needles to develop a multi-needle electrospinning setup [10,11]. Although the productivity of nanofibers could be improved in this way, some shortcomings of the multi-needle electrospinning setup still existed, such as difficulty in needle cleaning and the occurrence of corona discharge between the needles. Therefore, needleless electrospinning technology has been paid much attention as a new kind of method to produce nanofibers. The key issue was to design a spinneret that could simultaneously generate multiple jets for needleless electrospinning. All sorts of spinnerets were developed and investigated from 2004 up to present. Yarin and Zussman first proposed needleless electrospinning to form multiple nanofibers, and they used a two-layer system to generate steady vertical spikes on the upper layer of a polymer solution [12]. However, we found that this method produced less nanofiber from its typical optical image. A rotating cylindrical roller was developed to generate multiple jets for improving the productivity of nanofibers [13]. Currently, this electrospinning equipment named “Nanospider” has been reported and commercialized by the Elmarco company. In the following years, many electrospinning spinnerets were presented, such as rotary cone [14], metal roller [15], bubble [16,17], cylinder [18], disk [19], spiral coil [20], conical wire coil [21], stepped pyramid-shaped [22,23], multiple-ring [24], twisted wire [25], metal dish [26], needle-disk [27], and sprocket wheel disk [28]. Some important common issues were still found when these spinnerets were used to produce nanofibers, such as large area solvent volatilization, thick nanofiber diameter, and broad nanofiber diameter distribution. These problems have significantly impacted on the process of electrospinning and the quality of nanofibers. Therefore, a new spinneret still needs to be developed and investigated to overcome these problems. In recent years, our research group developed the annular spinneret, reducing solvent volatilization and making use of polymer solution [29,30]. Molnar used a shear-aided annular needleless electrospinning method to generate nanofibers, and the shearing effect could decrease the shear-thinning solution, which made it easy to form jet initiation [31]. These research works provided some novel ideas for further study to improve the optimization of the needleless electrospinning spinneret.

In this study, we presented a linear flume spinneret to fabricate nanofibers. Multiple jets could be formed on the top of the linear flume spinneret. Finite element analysis software was adopted to simulate the electric potential and the electric field distribution profiles. Both the electric potential value and the electric field intensity around the linear flume spinneret were also investigated. Later on, we further examined the effects of different spinning process parameters on the morphology and nanofiber diameter. The results demonstrated that small diameter and narrow distribution nanofibers could be achieved by adjusting the spinning process parameters. The average nanofiber diameter was 108 ± 26 nm. The average error was 24%. The productivity of nanofibers reached 4.85 ± 0.36 g/h, which was about 24 times more than that of the single-needle method. This linear flume spinneret has a great advantage to improve the quality and productivity of nanofibers. Moreover, this electrospinning setup has a significant potential for realizing the industrialization of nanofibers.

## 2. Experiment Details

### 2.1. Materials

Polyacrylonitrile (PAN) with a molecular weight of 75,000 g/mol was purchased from Sinopharm Chemical Reagent Co., Ltd. (Shanghai, China). *N,N*-Dimethylformamide (DMF) was purchased from Shanghai Lingfeng Chemical Reagent Co., Ltd. (Shanghai, China). PAN powder was dissolved in DMF to form polymer solution with three different concentrations (10 wt %, 11 wt %, 12 wt %). The PAN solutions were stirred on a magnetic stirrer for 24 h to form a homogeneous light-yellow transparent solution.

### 2.2. Needleless Electrospinning Apparatus

**Figure 1:**
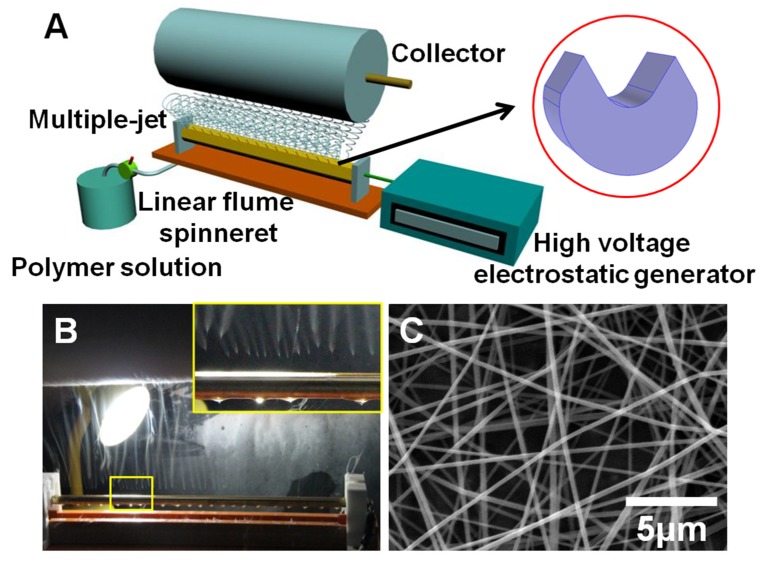
(**A**) Schematic of the multiple-jet needleless electrospinning setup, the shape of spinneret at the top right corner, (**B**) photograph of the linear flume spinneret spinning process, enlarged picture of multiple jet formation at the top right corner, (**C**) SEM image of nanofibers.

The apparatus of needleless electrospinning consisted of a linear flume spinneret, control pump, polymer solution, collector and a high voltage electrostatic generator, which are showed in Figure 1A. The polymer solution was transported to the linear flume spinneret. The solution flow rate of the polymer solution was accurately controlled by the control pump. The collector rotating at the speed of 70 r/min was used to collect generated nanofibers. Figure 1B shows the linear flume spinneret spinning process. Two rows of jets were formed simultaneously on the surface of the spinneret. An electrospinning video can be seen in the Supplementary Materials. The number of multiple jets was 2–3 jets/cm. A SEM image of nanofibers is showed in Figure 1C. We found that small and uniform distribution nanofibers could be fabricated successfully.

### 2.3. Characterization and Measurement

A scanning electron microscope (SEM, Quanta-250, Hillsboro, Oregon, USA) was used to observe the morphology of nanofibers. One hundred nanofibers in the SEM image were selected to measure the nanofiber diameter and diameter distribution using Image J software (v1.8.0, NIH, Bethesda, MD, USA). The productivity of nanofibers was weighted using electronic balance (FA2004A, Shanghai Jingtian Electronic Instrument Co., Ltd, Shanghai, China). The electric potential and the electric field distribution of the linear flume spinneret were simulated using the finite element analysis software entitled COMSOL Multiphysics 5.0 (COMSOL company, Stockholm, Sweden). A geometric model was established according to the actual size of the electrospinning setup. Mesh generation could be carried out with self-contained procedures. Electric potential and electric field distribution were displayed in the form of the contour.

## 3. Results and Discussion

### 3.1. Electric Field Simulation of the Linear Flume Spinneret

**Figure 2:**
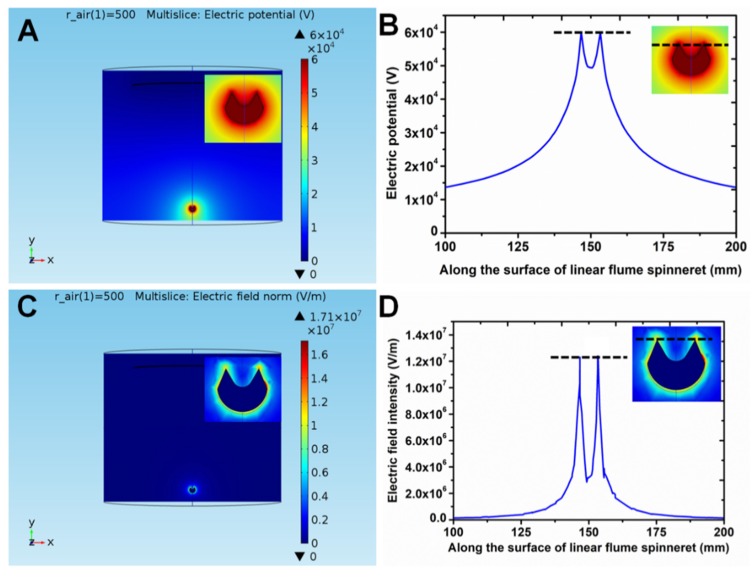
Electric potential and electric field of the linear flume spinneret. (**A**) The contour distribution and (**B**) curve of electric potential, the inset picture is an enlarge picture of the contour distribution of electric potential, (**C**) the contour distribution and (**D**) curve of electric field, the inset picture is an enlarge picture of the contour distribution of electric field. All black dotted lines represent the top of the linear flume spinneret.

The electric field of the needleless electrospinning spinneret played an important role in the formation of multiple jets during electrospinning. Therefore, we adopted finite element analysis software to simulate the electric potential and electric field intensity of the linear flume spinneret. Figure 2A–D shows the electric potential and electric field of the linear flume spinneret. It is obvious that intuitive changes in electric potential and electric field are found from the contour and curve. Figure 2A presents the electric potential contour of the linear flume spinneret with the applied voltage of 60 kV. The distance between spinneret and collector was 20 cm. The linear flume spinneret showed the highest electric potential, and then the electric potential gradually decreased. This situation can be seen clearly from the changes in color. The relationship between the surface of the linear flume spinneret and the electric potential was investigated, which is visualized by using curve mode in Figure 2B. Two peaks of the electric potential curve are found around 150 mm, which represent the two points of the highest electric potential, about 6 × 10^4^ V. In fact, the two points are two lines for the linear flume spinneret in three-dimensional space. The polymer solution was stored in the flume. Two rows of jets could be formed from the two peak lines of the spinneret when the electric force exceeded the join forces of surface tension force and viscosity resistance. Figure 2C shows the electric field distribution contour of the linear flume spinneret. The electric field intensity along the surface of the linear flume spinneret is examined in Figure 2D. We found that the two peak values of electric field intensity were 1.2 × 10^7^ V/m from the curve of the electric field intensity.

### 3.2. Effects of the Spinning Process Parameters on the Morphology of Nanofibers

**Figure 3:**
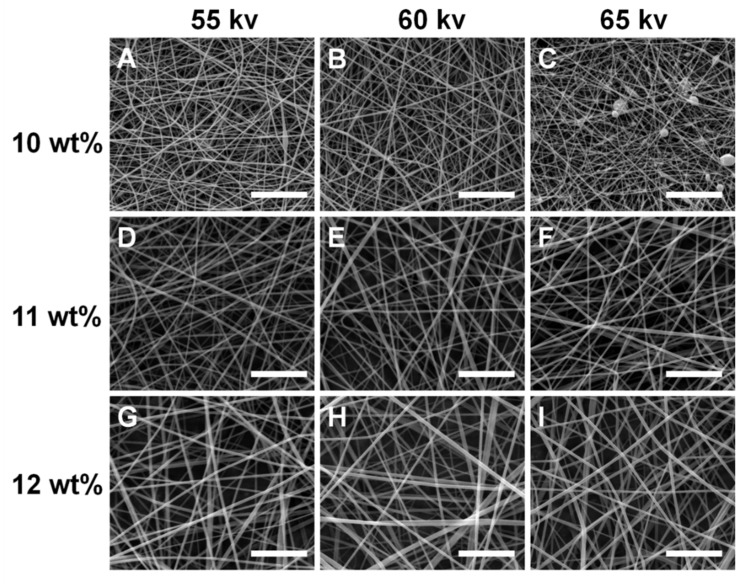
SEM images of nanofibers under different conditions of the spinning process. (**A**) 55 kV, 10 wt %; (**B**) 60 kV, 10 wt %; (**C**) 65 kV, 10 wt %; (**D**) 55 kV, 11 wt %; (**E**) 60 kV, 11 wt %; (**F**) 65 kV, 11 wt %; (**G**) 55 kV, 12 wt %; (**H**) 60 kV, 12 wt %; (**I**) 65 kV, 12 wt %. Scale bar = 5 μm.

Figure 3 shows the effects of spinning process parameters on the morphology of nanofibers when the collection distance is 15 cm. Three different solution concentrations and three different applied voltages were selected to fabricate nanofibers. Nine SEM images were obtained to compare the features of nanofibers. Figure 3A–C presents the effects of different applied voltages on the morphology of nanofibers when the solution concentration was 10 wt %. It is obvious that finer nanofibers could be fabricated. The reason was that the solution concentration of 10 wt % was relatively low. Some bead nanofibers can be seen in Figure 3C when the applied voltage is 65 kV. This was because a higher electric force acted on the low concentration spinning solution to bring more solution volume within a certain time. Meanwhile, the greater solution volume did not have enough time to form nanofibers under the condition of the higher electric force. Meanwhile, the travel time to the collector was now shorter, hence less evaporation time. Thus, bead nanofibers might be produced during electrospinning. Figure 3D–F shows the effect of different applied voltages on the morphology of nanofibers when the solution concentration was 11 wt %. The morphology of nanofibers became coarse compared with the solution concentration of 10 wt %. We found that no bead nanofibers could be fabricated successfully. Figure 3G–I exhibits the effect of different applied voltages on the morphology of nanofibers when the solution concentration was 12 wt %. The morphology of nanofibers became thicker compared with the solution concentration of 11 wt %. Therefore, it is very important for us to select the suitable solution concentration and applied voltage for preparing the desirable morphology and diameter of nanofibers.

### 3.3. Effects of the Spinning Process Parameters on Nanofiber Diameter

**Figure 4:**
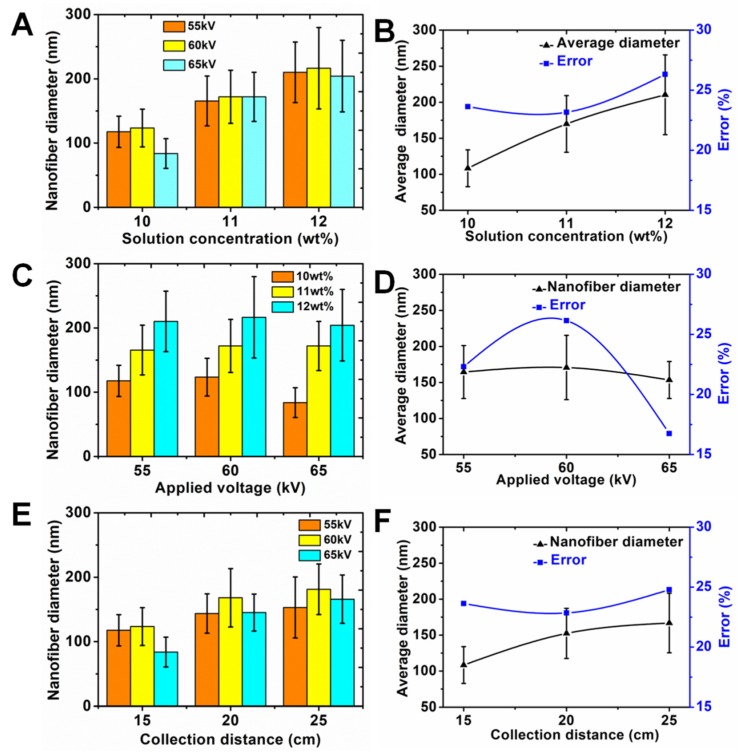
Effect of solution concentration on (**A**) nanofiber diameter and (**B**) average diameter and error. Effect of applied voltage on (**C**) nanofiber diameter and (**D**) average diameter and error. Effect of collection distance on (**E**) nanofiber diameter and (**F**) average diameter and error.

The spinning process parameters including solution concentration, applied voltage and collection distance had a direct influence on the nanofiber diameter. Figure 4 shows the experiment results. Figure 4A presents the effects of the different solution concentrations (10, 11 and 12 wt %) on the nanofiber diameter. In order to investigate the nanofiber diameter more accurately, three different applied voltages (55, 60 and 65 kV) were used to fabricate nanofiber membranes for each solution concentration. As shown in Figure 4B, the average nanofiber diameter and average error could be calculated according to three different nanofiber membranes by using three different applied voltages for each solution concentration. The average nanofiber diameters were 108 ± 26, 170 ± 39 and 210 ± 55 nm, and the average errors were 24%, 23% and 26% for solution concentrations of 10, 11 and 12 wt %, respectively. We found that the average nanofiber diameter gradually increased with the increasing of solution concentration. A high solution concentration led to more macromolecular chain entanglement, so the nanofiber diameter increased. Here, the average error was an important index to evaluate the dispersion degree of nanofibers, which could reflect the quality of nanofibers. Low error represented a small dispersion degree, which demonstrated a high quality of nanofibers. The results showed that the average error increased with the higher solution concentrations. Therefore, it was very important for improving the quality of nanofibers to adjust to an appropriate solution concentration. Figure 4C shows the effect of applied voltage on the nanofiber diameter under the condition of three different solution concentrations. As for each applied voltage, we found that the nanofiber diameter gradually increased with the increasing of solution concentration. Figure 4D presents the results of average nanofiber diameter and average error with the increasing of applied voltages. The average nanofiber diameters were 164 ± 37, 171 ± 45 and 153 ± 26 nm, and the average errors were 23%, 26% and 17% for applied voltages of 55, 60 and 65 kV, respectively. We found that the average nanofiber diameter first increased and then decreased with the increasing of applied voltage. This may be explained by the interaction between electrostatic force and viscous resistance. The results indicated that the applied voltage had a different impact on the nanofiber diameter compared with the solution concentration. It should be noted that the average error first increased and then decreased with the increasing of applied voltage. The error was only 17% when the applied voltage was 65 kV. This result proved that a feasible method to improve the quality of nanofibers was to use a higher voltage to fabricate nanofibers. The effect of collection distance on nanofiber diameter under the condition of three different applied voltages is showed in Figure 4E. For each collection distance, the nanofiber diameter first increased and then decreased with the increasing of collection distance. Figure 4F exhibits the results of average nanofiber diameter and average error with the increasing of collection distances. The average nanofiber diameters were 108 ± 26, 152 ± 35 and 167 ± 41 nm, and the average errors were 24%, 23% and 25% for collection distances of 15, 20 and 25 cm, respectively. It was found that the average nanofiber diameter gradually increased with the increasing of collection distance. The reason was that increasing the collection distance weakened the electric field intensity between the linear flume spinneret and collector. Those jets were not adequately stretched, therefore, the nanofiber diameter became thicker. The average error for nanofiber diameter showed little change with the increasing of collection distances. The error was only 23% when the collection distance was 20 cm. Except for solution concentration and applied voltage, collection distance was also an important parameter and it should be considered during needleless electrospinning.

### 3.4. Effects of the Spinning Process Parameters on the Productivity of Nanofibers

**Figure 5:**
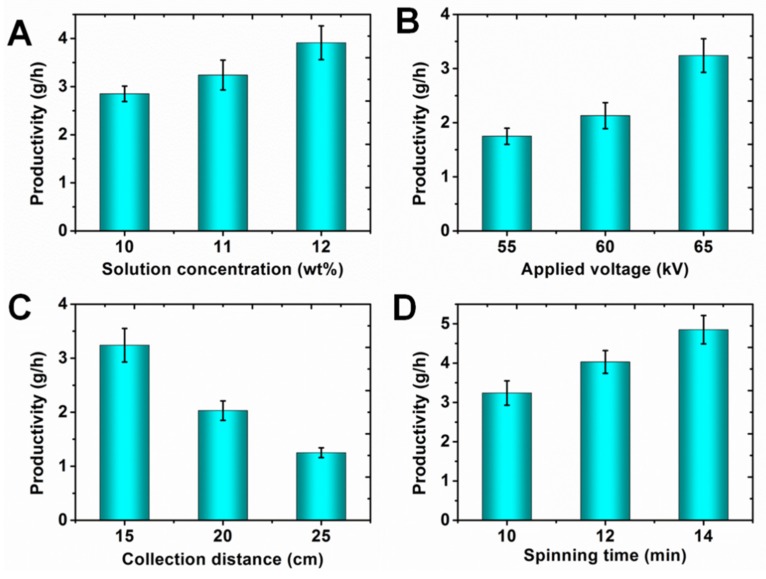
Effects of different process parameters on the productivity of nanofibers: (**A**) solution concentration; (**B**) applied voltage; (**C**) collection distance; (**D**) spinning time.

Compared with the single-needle electrospinning setup, the linear flume spinneret needleless electrospinning approach could significantly enhance the productivity of nanofibers. We investigated the effect of different spinning process parameters on the productivity of nanofibers. Figure 5A shows the influence of solution concentration on the productivity of nanofibers. It is obvious that the productivity of nanofiber increased with the increasing of solution concentration. The reason was that increasing the nanofiber diameter improved the productivity. The productivity of nanofibers reached 3.91 ± 0.35 g/h. The impact of the applied voltage on the productivity of nanofibers is showed in Figure 5B. The productivity of nanofibers also increased with the increasing of applied voltage. This was because that more jets could be formed under the higher applied voltage. We also examined the effect of collection distance on the productivity of nanofibers. In contrast with solution concentration and applied voltage, the productivity of nanofibers decreased dramatically with the increasing of collection distance. The increase in collection distance caused the lowering of the electric field intensity of the linear flume spinneret, which could reduce the number of multiple jets. In addition, the increased collection distance also led to more nanofibers being able to drift away in the air. Therefore, the productivity of nanofibers decreased with the increasing of collection distance. The spinning time also played an important role in improving the productivity of nanofibers. Figure 5D presents the influence of the spinning time on the productivity of nanofibers. The productivity of nanofibers was 4.85 ± 0.36 g/h when the spinning time was 14 min, which was about 24 times more than that of the single-needle method. Compared with the single-needle electrospinning setup, the spinning efficiency could be greatly improved with the linear flume spinneret needleless electrospinning setup.

## 4. Conclusions

In this study, we developed a novel linear flume spinneret to produce multiple jets and fabricate nanofibers by needleless electrospinning technology. Multiple jets with two rows were generated from the surface of the linear flume spinneret. The electric potential and electric field intensity of the linear flume spinneret were investigated. The effects of different spinning process parameters on the nanofiber diameter and error were examined in detail. The productivity of nanofibers was calculated with the different spinning process parameters. The average diameter of nanofibers was controlled between 108 and 210 nm, and the average error of nanofibers was controlled between 17% and 26%. The productivity of nanofibers reached 4.85 ± 0.36 g/h, which was about 24 times more than that of the single-needle method. Therefore, high-quality nanofibers could be fabricated by adjusting the spinning process parameters, and this novel linear flume spinneret has great potential for producing high-quality and high-productivity nanofibers.

## Supplementary Materials

The following are available online at https://www.mdpi.com/2073-4360/11/12/2052/s1.
